# UV-Green Iridescence Predicts Male Quality during Jumping Spider Contests

**DOI:** 10.1371/journal.pone.0059774

**Published:** 2013-04-03

**Authors:** Matthew L. M. Lim, Daiqin Li

**Affiliations:** 1 Department of Biological Sciences, National University of Singapore, Singapore; 2 College of Life Sciences, Hubei University, People’s Republic of China; The Australian National University, Australia

## Abstract

Animal colour signals used in intraspecies communications can generally be attributed to a composite effect of structural and pigmentary colours. Notably, the functional role of iridescent coloration that is ‘purely’ structural (i.e., absence of pigments) is poorly understood. Recent studies reveal that iridescent colorations can reliably indicate individual quality, but evidence of iridescence as a pure structural coloration indicative of male quality during contests and relating to an individual’s resource-holding potential (RHP) is lacking. In age- and size-controlled pairwise male-male contests that escalate from visual displays of aggression to more costly physical fights, we demonstrate that the ultraviolet-green iridescence of *Cosmophasis umbratica* predicts individual persistence and relates to RHP. Contest initiating males exhibited significantly narrower carapace band separation (i.e., relative spectral positions of UV and green hues) than non-initiators. Asymmetries in carapace and abdomen brightness influenced overall contest duration and escalation. As losers retreated upon having reached their own persistence limits in contests that escalated to physical fights, losers with narrower carapace band separation were significantly more persistence. We propose that the carapace UV-green iridescence of *C. umbratica* predicts individual persistence and is indicative of a male’s RHP. As the observed UV-green hues of *C. umbratica* are ‘pure’ optical products of a multilayer reflector system, we suggest that intrasexual variations in the optical properties of the scales’ chitin-air-chitin microstructures are responsible for the observed differences in carapace band separations.

## Introduction

Structural coloration is a product of the selective reflection of a light spectrum due to the presence of optically functional surface nanostructures [1]. Of recent interest to optical and behavioural scientists is a kind of structural coloration known as animal reflectors [2] or iridescence. Popular examples of such multilayer interference reflectors include the alternating chitin-air layers of lepidopteran iridescent scales [3], the stacks of protein plates interspersed by cytoplasm spaces in cephalopod iridophore cells [4], and the alternating laminar membranes of a stomatopod’s reflecting cuticle [5]. These multiple thin-film reflectors are postulated to have key functional roles in intraspecific communication and in the evolution of iridescent coloration [6].

Though the composite effects of pigmentary and structural hues in animal colorations are increasingly being studied (e.g., [7], [8]; also see [1]), animal iridescence that is purely structural (i.e., absence of pigments) has also received some attention (e.g. [9], [10], [11], [12]). Animal iridescence is already known to reliably inform of an individual’s condition, health and age (e.g. [10], [11], [13]. However, questions on whether ‘pure’ iridescent colorations can reliably indicate an individual’s condition or status during intraspecies communication have ignited explorations to understand the specific mechanisms and proximate factors responsible for the observed inter- and intrasexual variations in iridescence. For example, the iridescent colorations of the male Anna’s hummingbird *Calypte anna* is condition-dependent and influenced by a protein-poor diet that consists mainly of sugars [11]. Also, the blue-green iridescence of the male damselfly *Calopteryx maculata* advertises its territorial status to conspecific male rivals, with blue (i.e., fatter) males postulated to be more territorial than green (i.e., leaner) individuals [14] (but also see [15]). It is proposed that the observed among-male variations (i.e., hue shifts) in abdomen iridescence correlates with prey amount, where alterations of the lamella distance results in short- (i.e., blue) or long wavelength (i.e., green) colour shifts [14]. If ‘pure’ structural colours can advertise an individual’s quality during contests, relating iridescence to an individual’s resource-holding potential (RHP [16]) in resolving conflicts is possible. Still, though many animal colorations do consist of a structural component [17], many structural colorations (e.g., UV-structural colorations) investigated in male-male conflicts are not ‘purely’ structural; this may obscure accurate correlative analyses of animal iridescence and the RHP of an individual (e.g. [18], [19], [20]).

During a contest, competitors are assumed to minimize potential costs by mutually assessing each others’ strengths and weaknesses, such that contests with higher asymmetries are resolved faster [16]. RHP relates the outcome of pair-wise conflicts with differences in individual quality. This is linked to asymmetric phenotypic traits that can influence contest dynamics and outcomes, for example body size and colour patches [21], [22]. Injuries and fatalities [23], [24] associated with escalated contests can reduce individuals’ likelihoods of winning and hence are avoided only if conflicts can be resolved sooner (rather than later) and rivals can assess the probability of winning before a visual encounter escalates to relatively more costly physical fights. Hence it is assumed that rivals assess each others’ RHP in the form of phenotypic traits to reliably identify an individual’s quality relative to their own. However, a recent argument highlighted that many contests are possibly resolved by self assessments (i.e., self RHPs) rather than mutual assessments of each other’s RHPs ([25]; for a review see [26]), with contest duration solely dependent on the loser’s RHP. This means that the time taken to resolve a conflict is entirely dependent on the RHP of the loser or the weaker rival, where the contestants’ persistence are in accordance with their own RHP. While conflict resolution via self-assessments of RHP using body mass has been established (i.e., strong prediction from the relative mass of losers to contest duration [25]), little is known about the role of structural coloration differences in resolving conflicts.

To investigate whether iridescence influences conflict resolution and relates to individual RHP, we examine whether male-male contests in the UV-green iridescence jumping spider *Cosmophasis umbratica* (Simon 1903) [27] are resolved by self- or mutual assessments. We propose that salticids are ideal study models because many species exhibit iridescent colorations (e.g. [28]), and all generally possess a colour vision system that extends into the short wavelengths [29] and exhibit elaborate vision-mediated threat displays [30]. Indeed, *C. umbratica* is an ideal model since the short wavelength component of its UV-green iridescence is known to be condition-dependent [31] and crucial for intraspecific communications [32], [33], [34]. In most male-male *C. umbratica* interactions, competing rivals exhibit a repertoire of displays (e.g., hunched legs, grappling and pushing [27]) that occur in a temporal sequence (i.e., visual to tactile to physical), with a progression of increasing proximity (i.e., decreasing distance between rivals), rising energy expenditure and increasing risk of injury (fig. S1). Perhaps, most importantly, the UV-green iridescence [35] of *C. umbratica* is known to be purely structural [12], an optical effect of a chitin-air-chitin ‘air cushion’ multilayer design ([12]). In addition, as a male-male conflict usually consists of a visual and a physical phase in this species [27], we analyzed the durations of the physical phase (i.e., physical contest) separately from the overall contest duration (i.e., sum of the durations of the visual and physical phases). We excluded the period of visual (i.e., non-physical) interactions in defining the duration of physical contest so as to relate an individual’s physical endurance or persistence to the weaker (i.e., loser) contestants’ RHP during the more energetic-demanding physical phase [36]. We also relate overall contest duration to contest escalation since both variables are significantly correlated (see Results). By log-transforming overall contest and physical contest durations (i.e., response variables), we investigate the relationship between contest duration (loser persistence) and the spider’s UV-green iridescence. We ask whether conflicts between age- and size-controlled adult males are resolved by self- or mutual assessment [26] using separate multiple regression analyses, and if the iridescent colour traits relates to individual RHP.

## Materials and Methods

### (a) Ethics Statement

No specific permits were required for the described studies, as these jumping spiders were neither protected nor endangered species. Sub-adult males were collected from re-growth *Ixora javanica* along a public cycling route at Ulu Pandan Park Connector, Singapore. We conducted our laboratory experiments in accordance with the ‘Principles of Animal Care’ (publication No. 86-23, revised 1985) of the National Institute of Health, and with the Institutional Animal Care and Use Committee of the National University of Singapore.

### (b) Study Species

Sub-adult males (i.e., one molt away from adult) of *C. umbratica* were individually housed and maintained that followed earlier protocols [31], [32]. We sought to minimize the effects of age, body size (i.e., carapace length (mean ± SD: 25.615±3.750 mm), abdomen length (mean ± SD: 37.885±5.745 mm) and mass (mean ± SD: 0.0157±0.0064 g)) as proximate factors that may influence contest dynamics and outcomes. We paired size-matched males (N = 26) to the best of our abilities, while also ensuring that paired rivals were of comparable age (i.e., did not moult more than 5 days apart). Hence, all adult virgin males used (n = 52) were of comparable post-maturation age (i.e., males within 2 weeks of age), body size and mass with no prior contest experience.

### (c) Reflectance Spectrometry

Though several body parts are displayed during intraspecific interactions [27], we chose to focus on abdomen and carapace colorations because these exhibited relatively greater intrasexual colour variations [35] and are more prominently displayed in male-male interactions [27]. Reflectance spectra of the carapace and abdomen, measured using an Ocean Optics USB2000 spectrometer, yielded the following [31]: (i) UV intensity (*R*_UV_: % reflectance of UV reflection band (λ_300–400 nm_)); (ii) intensity of human-visible (VIS) wavelengths (*R*_VIS_: % reflectance of VIS reflection band (λ_400–700 nm_)); (iii) total intensity (*R*_total_: area under reflectance curve (λ_300–700 nm_)); (iv) UV hue (λ(*R*_UV_): spectral position of UV band peak (i.e., wavelength of maximal reflectance in UV range (300–400 nm)); (v) VIS hue (λ(*R*_VIS_): spectral position of VIS band peak (i.e., wavelength of maximal reflectance in VIS range (400–700 nm)); and (vi) band separation (λ_VIS-UV_: wavelength difference (in nm) between UV and VIS hues). Band separation [31] defines the relative spectral positions of UV and green hues, the main and side reflection bands of a multilayer optical system [2] in this species [12]. Since both UV and VIS intensities did not exhibit significant differences between winners and losers as well as between initiators and non-initiators (Supporting Information Table S1 & S2), and that both traits are predictably positively correlated due to their relationship as side- and main reflection bands of a multilayer reflector [2], [12], we relied on total intensity (i.e., sum of UV and VIS intensities) for data analyses.

### (d) Behavioral Experiments

We performed male-male contests (n = 26) from an open stage (i.e., a horizontal A4 rigid white paper held 30 cm above the experimental table by a vertically-standing paper roll); this removed any biases of rivals based on their relative positions on vertical walls in an enclosed stage. A black opaque curtain that surrounded the experimental table minimized disturbances during video recording of experiments. Ten Voltarc Ultra Light tubes (110 W each) held 100 cm above the stage provided a constant and full-spectral (300–700 nm) illumination [34]. Two males, held individually in Petri dishes (diameter: 3 cm; height: 1 cm), were then placed 12 cm apart on the stage. An opaque black self-standing cardboard screen placed between the dishes (i.e., 6 cm from either Petri dish) prevented visual contact between rivals during the acclimatization period (5 min) with full spectral lights being turned on. A contest trial began when both Petri dishes and the cardboard were removed, and when one male initiated a contest by displaying agonistic behavior (‘hunched’ posture [27]) towards a conspecific rival male, and ended when one male retreated (‘decamped’ [27]). Each trial was allowed to repeat a maximum of three times if no agonistic interactions occurred or any individual leapt away from the stage before any visual or agonistic interactions occurred. If there was still no interaction the trial was considered void and excluded from data analysis. We video recorded all contests using a high-definition digital video camera (Sony HDV 1080i).

We noted the following variables from slow-motion frame-by-frame playbacks (Studio DV Plus, Pinnacle Systems Inc., Mountain View, CA) of contest videos filmed at 30 frames per second: 1) winner (i.e., the male that did not ‘decamp’ at the end of agonistic interaction); 2) loser (i.e., the male that ‘decamped’ during or at the end of an agonistic interaction); 3) initiator (i.e., the individual that displayed agonistic behavior first); 4) non-initiator (i.e., the individual that did not display agonistic behaviour first or failed to display any agonistic behaviour); 5) physical contest duration (i.e., the time elapsed from the point when rivals commenced to push and grapple and eventual escalation to struggle and bite until one male decamped). We classified all bouts based on escalation levels, using different colours to associate with each level. Level 1 (blue): one or both individuals adopted hunched position but with no body contact (level 1; blue); level 2 (yellow): both males clashed briefly; level 3 (orange): both males engaged in a physical battle of grappling and pushing (fig. S1); level 4 (red): physical and violent conflict of both males engaged in a non-orderly fashion of rolling and biting [27]. Each male was used once throughout the experiment.

### (e) Data Analyses

To ensure minimal influence of morphological asymmetries on contest dynamics and outcomes, we first compared the morphological traits (i.e., body length and mass) of winners and losers, and of initiators and non-initiators, using paired *t*-tests or Wilcoxon signed-ranks test if the data were not normally distributed (Kolmogorov–Smirnov test; IBM SPSS Statistics v19). We did not analyze these traits further because the mass and length of individuals did not appear to influence contest initiation and outcomes (tables S1, S2). We then used linear regression analysis to examine the effects of colour asymmtery (i.e., winners – losers) on log-transformed physical contest duration and log-transformed overall contest duration and escalations. This was followed by multiple regression analysis to ascertain whether these contest variables were predicted by either or both winners’ and losers’ colour traits as covariates [37]. Because band separation is expressed as the wavelength difference of VIS and UV hues, we investigated the effect of UV and VIS hues of winners and losers on contest dynamics, using the spectra positions of both hues as covariates in a multiple regression analysis (IBM SPSS Statistics v19).

## Results

**Figure 1:**
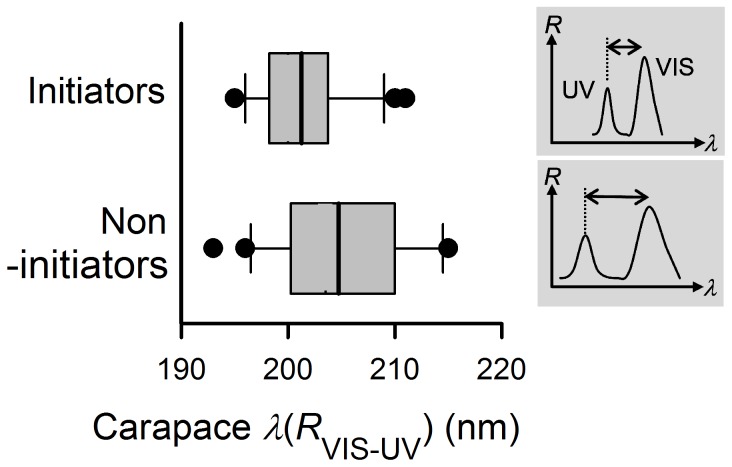
Differences in carapace band separations of contest initiators and non-initiators. Initiating males exhibited narrower band separations as compared to non-initiative males. Inserts are illustrations of *Cosmophasis umbratica* reflectance spectra with variations in carapace band separation between initiators (top) and non-initiators (bottom). Central bar: mean; hinges: 25 and 75%; whiskers: 5 and 95%; circles: outside values. *R*: reflectance (%); *λ*: wavelength (nm).

We found that contest initiators exhibited significantly narrower carapace band separation (i.e., combination of a long wavelength shift in UV hue and a short wavelength shift in green hue) as compared to non-initiators (fig. 1); no other colour traits differed significantly (table S1). We also found no significant differences in any morphological and colour traits between winners and losers (table S2).

**Figure 2:**
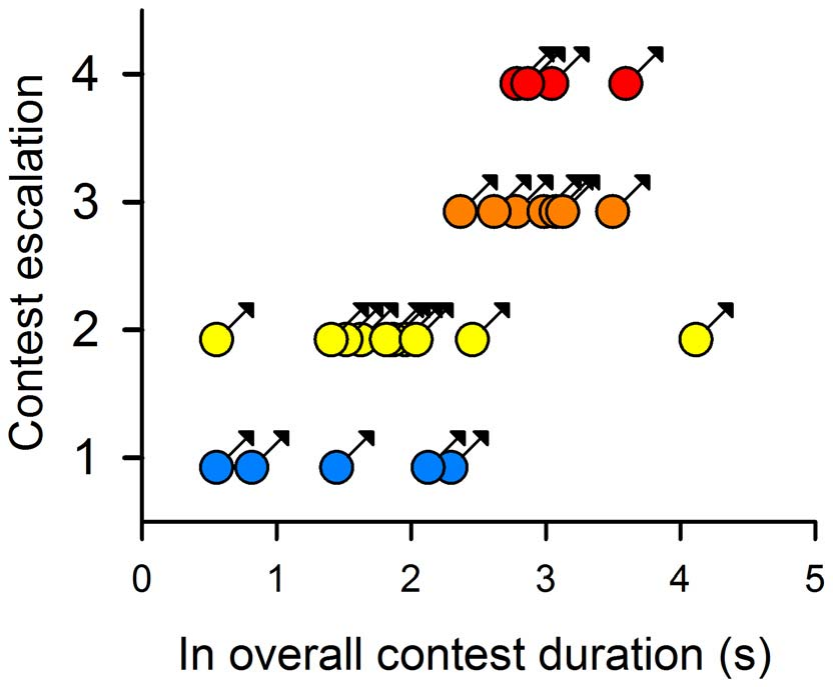
Correlation of overall contest duration and contest escalation. Contest durations (natural log) and escalation are highly correlated (Spearman’s Rank Order correlation: *r* = 0.72, P = 0.00000285). Coloured symbols (blue, yellow, orange and red) relate to escalation levels (1, 2, 3 and 4 [27]) from least (blue) to most (red) energy-demanding contests.

**Figure 3:**
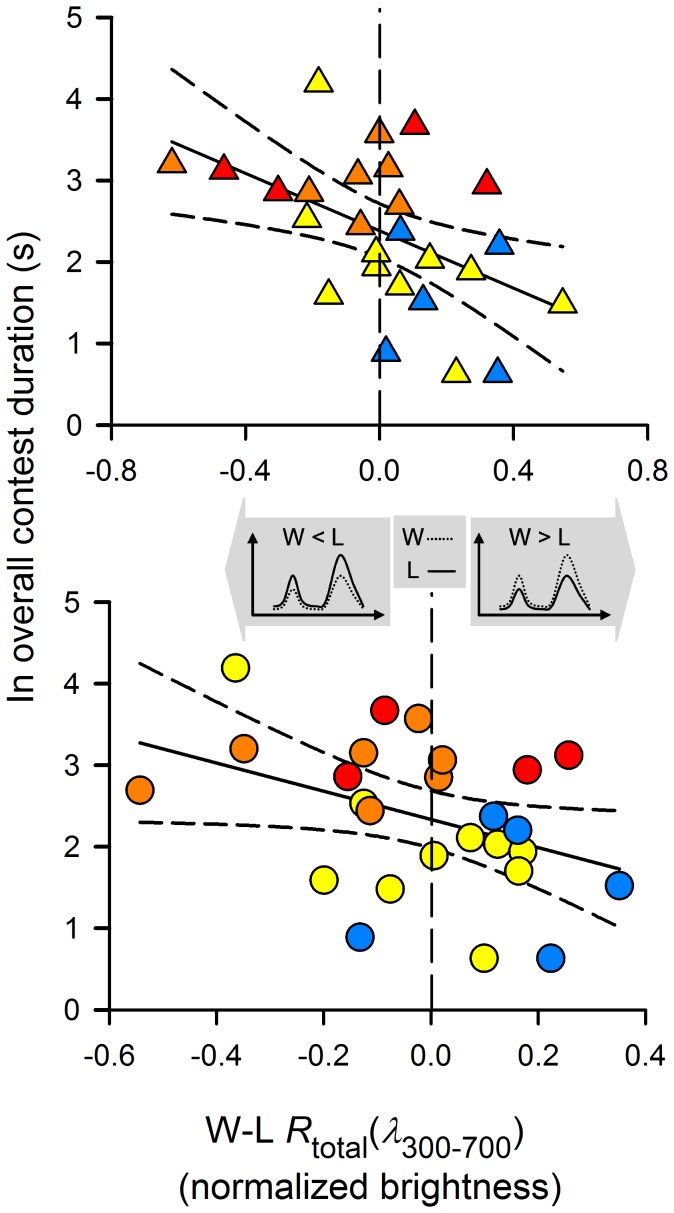
Asymmetries (W−L) in carapace (Δ; top) and abdomen (○: bottom) total brightness predicting overall contest duration and escalation. Negative slopes indicated that non-physical bouts (i.e., blue and yellow symbols) are dictated by high, positive asymmetry in body total brightness (i.e., winners exhibit brighter carapace than losers, W>L). Contests escalated as positive asymmetry decreases and approaches zero (i.e., vertical dashed lines). Negative effect of rival asymmetry in carapace total brightness on overall contest duration and escalation (top) is further explained by negative and positive effects of winners’ and losers’ carapace total brightness, respectively (see table S3 and fig. S2). Shaded arrows indicate direction of variation in total brightness (less bright or brighter). Complete lines: best fit lines; dashed lines: 95% confidence intervals.

**Figure 4:**
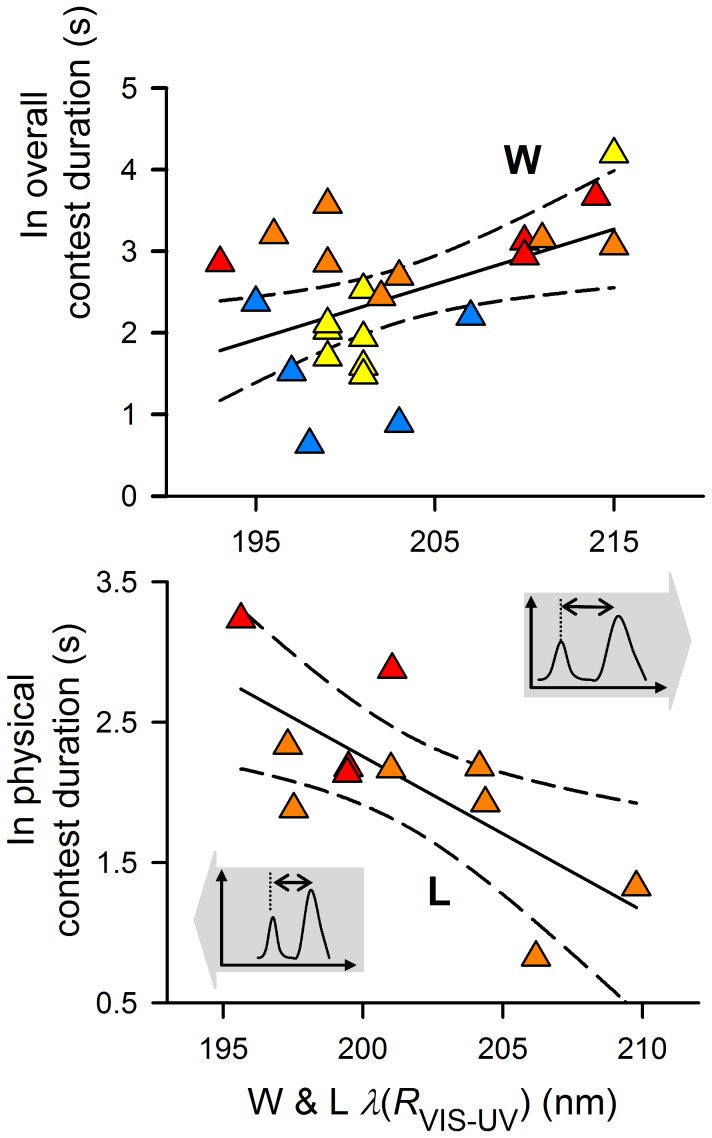
Winners’ and losers’ carapace band separations predicting overall contest duration and physical duration. Winners’ (W; top) carapace band separation predicted overall contest duration and escalation (but see table S3 and fig. S3, S4). Losers’ (L; bottom) carapace band separation predicted physical contest duration, with smaller carapace band separation significantly related to higher individual persistence (but see Table S4 and fig. S5). Shaded arrows indicate direction of variation in band separation (narrower or broader). Complete lines: best fit lines; dashed lines: 95% confidence intervals. Inserts are illustrations of *Cosmophasis umbratica* reflectance spectra with variations in carapace band separation between more persistent (narrower band separation; left) and less persistent (less narrow band separation; right) individuals. *R*: reflectance (%); *λ*: wavelength (nm).

Contest duration was significantly and positively correlated with contest escalation (fig. 2); visual contests that lasted longer escalated to bouts characterized by physically more robust male-male agonistic behavioral traits [27]. Rival asymmetry (i.e., winners − losers; W−L) in both carapace and abdomen total brightness had a significant negative effect on overall contest duration and escalation (fig. 3 and table S3 but see fig. S2). Narrower carapace band separation of winners had a significant negative effect on overall contest duration and escalation (fig. 4 and table S3 but see fig. S3 and S4); bouts that involved rival pairs with smaller differences in carapace band separation persisted longer (table S4 and fig. S5). These bouts escalated to the physical phase, with the persistence of physical contest attributed to losers but not winners: individuals with narrower carapace band separation persisted significantly longer (fig. 4). Contest persistence was dependent on the wavelength difference (i.e., band separation) between, rather than individual, spectral hues of UV and green (table S4).

## Discussion

Our results show that the UV-green iridescence of *C. umbratica* influenced overall contest duration, escalation, and the duration of the physical contests. Differences in rivals’ total brightness and winners’ carapace band separation significantly influenced contest duration and escalation. Losers’ carapace band separation predicted the duration of physical contests; the relatively higher (but negative) standardized regression coefficients of losers’ carapace band separation (losers: −0.101±0.037; winners: 0.022±0.020) further supports this (table S4; also see [38]). Both contest initiators and losers’ persistence were characterized by smaller carapace band separations. Together, these results support our postulation on the role of ‘pure’ structural coloration in conflict resolution, where the UV-green iridescent coloration of *C. umbratica* males do relate to an individual’s contest persistence and RHP. It is, however, of interest to note that rivals with smaller carapace band separation did not influence contest outcomes (i.e., individuals with smaller carapace band separation were not necessarily winners). A possible explanation is that contest duration, rather than contest outcome, may offer a more accurate perception of the motivational variables underlying contest behaviour and hence allows stronger statistical analyses for detecting any effects (e.g., [39]). Future contest studies on correlations between individual persistence and the colour traits of *C. umbratica* should consider the presence of a female as a motivation, and using males of similar sizes across all pairwise contests.

We expected rival *C. umbratica* males to display colour traits indicative of an individual’s persistence or RHP when resolving a conflict. Earlier studies have indicated that the UV-green iridescence of *C. umbratica* relates to age and body conditions. Well-fed adult males reflected brighter abdomen colorations than when starved [31]; younger (i.e., recently molted) adult males exhibited more long wavelength-shifted UV hues (i.e., hence narrower band separation) and have brighter carapace colorations than presumably older wild-caught adult males [31]. We do not assume these as proximate factors in our study since we tried our best to minimize age and diet asymmetries (i.e., only recently molted adults were used, and all sub-adults collected had similar feeding regime). Our approach (i.e., using rivals of similar size) may have reduced the statistical power to detect the effects of body size on contests in this species [38]; however, we found no correlations between body mass and any carapace colour traits (fig. S6) even though an expected positive correlation between abdomen total brightness and mass is observed since large males can possibly emulate well-fed males in abdomen brightness (see [31]). In contest resolution between size- and age-controlled rivals, we found that only carapace UV-green iridescence (i.e., band separation) differed significantly between initiators and non-initiators. Initiators exhibited significantly smaller carapace band separation as compared to those of non-initiators. Interestingly, an earlier contest study that used pairwise salticid males of different sizes also revealed that initiators were eventual winners [36]. In our study, losers with smaller carapace band separation persisted longer. Collectively, both results (i.e., that carapace band separation relates to contest intiation and loser’s persistence) strengthen the postulation that carapace UV-green iridescence indeed predicts an individual’s persistence and RHP during a contest.

Because the iridescent coloration of *C. umbratica* are purely an optical effect of multilayer interference, an optical system that totally lacks pigments [12], we propose that optical factors are solely responsible for intersexual variations in carapace band separation and total brightness. The highly prominent UV-green iridescence of adult *C. umbratica* males are due to numerous minute transparent scales, with each scale comprised of a dimension-specific chitin-air-chitin sandwich. These scales collectively produce the salticid’s characteristic UV and green hues [12]. As variations to the optical layers’ refractive index and optical thickness can influence brightness and the spectral positions of both UV and green hues (i.e., variations in band separation) [12], it is most likely that among-male variations in carapace total brightness and the relative spectral positions of UV and green hues, colour traits that relate to RHP, are directly related to variations of one or a combination of the above mentioned optical factors. Although the role of UV hues during conspecific interactions is known [12], [32], [33], [34], we know nothing about the function of green hue. Perhaps, only when both hues (i.e., UV-green iridescence) are interpreted together (i.e., band separation) will the role of UV-green iridescence in communication be better understood. Nonetheless, our findings here (i.e., UV-green iridescence predicts individual persistence during conflict resolution) may have bearings on future studies investigating the role of ‘pure’ iridescent colorations in animal communications.

## Supporting Information

Figure S1 **Movie on contest escalation of** ***Cosmophasis umbratica*** **males.**
(WMV)

Figure S2 **Influence of winners’ (W; top) and losers’ (L; bottom) carapace (Δ) total brightness on overall contest duration (natural log).** Complete lines: best fit lines; dashed lines: 95% confidence intervals. Coloured symbols (blue, yellow, orange and red) relate to escalation levels (1, 2, 3 and 4) from least (blue) to most (red) energy-demanding contests.
(TIF)

Figure S3 **Influence of winners-losers asymmetry (W−L) in carapace (Δ) band separation on overall contest duration (natural log).** Coloured symbols (blue, yellow, orange and red) relate to escalation levels (1, 2, 3 and 4) from least (blue) to most (red) energy-demanding contests.
(TIF)

Figure S4 **Influence of winners-losers asymmetry (W−L; top) and winners’ (W; bottom) carapace (Δ) VIS hue on overall contest duration and escalation, with presence (left) and absence (right) of data points where leverage values exceeded leverage critical values (circled points).** Both rival asymmetry (*R* ^2^ = 0.13; *P* = 0.084) and winners’ (*R* ^2^ = 0.153; *P* = 0.066) carapace VIS hue did not predict overall contest duration and escalation after outliers (circled symbols) were removed. Outliers were identified using leverage critical values from the formula (3*p*−1)/*n*, where *p* and *n* refer to number of parameters and sample size, respectively. Coloured symbols (blue, yellow, orange and red) relate to escalation levels (1, 2, 3 and 4) from least (blue) to most (red) energy-demanding contests.
(TIF)

Figure S5 **Influence of asymmetry (W−L) in carapace (Δ) band separation on duration of physical contests.** Coloured symbols (orange and red) relate to escalation levels 3 and 4, respectively.
(TIF)

Figure S6 **Correlations of carapace (Δ) or abdomen (○) colour traits with body mass of all individuals.** Only abdomen total brightness exhibited a positive correlation with body mass. R: Spearman’s correlation coefficient.
(TIF)

Table S1 **Differences in morphological and colour traits between initiators and non-initiators.**
(DOCX)

Table S2 **Differences in morphological and colour traits between winners and losers.**
(DOCX)

Table S3 **Effects of carapace and abdomen colour traits on log-transformed overall contest duration.** L, W, and W-L denote losers, winners, and winner-loser asymmetry, respectively.
(DOCX)

Table S4 **Effects of carapace and abdomen colour traits on log-transformed overall duration of physical contests.** L, W, and W-L denote losers, winners, and winner-loser asymmetry, respectively.
(DOCX)
