# Supplementary figures and images for: UV-Green Iridescence Predicts Male Quality during Jumping Spider Contests
Source: PLoS One. 2013 Apr 3;8(4):e59774. doi: 10.1371/journal.pone.0059774 (PMC3616068; doi:10.1371/journal.pone.0059774)

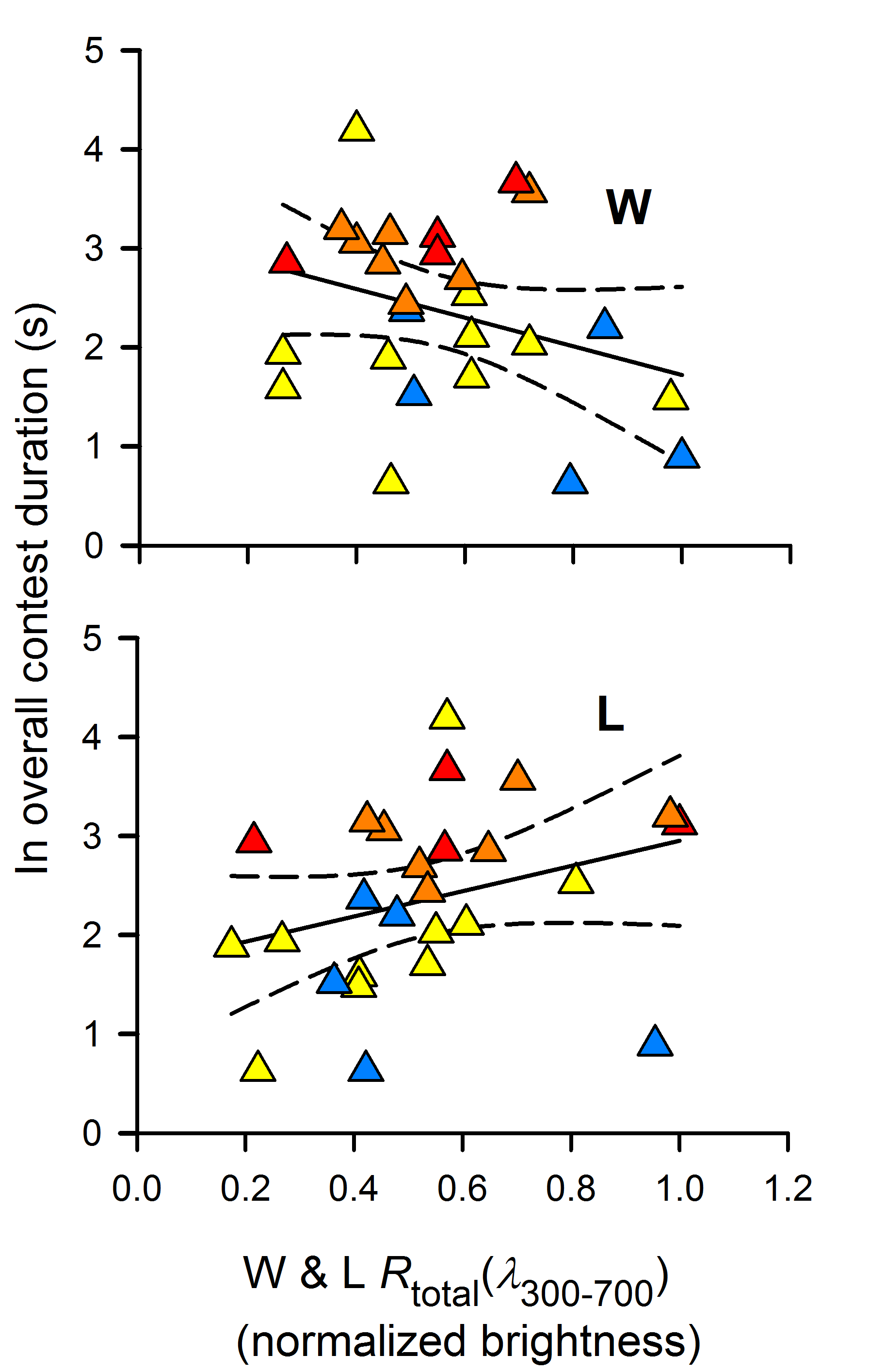

Supplement: Figure S2 — Influence of winners’ (W; top) and losers’ (L; bottom) carapace (Δ) total brightness on overall contest duration (natural log). Complete lines: best fit lines; dashed lines: 95% confidence intervals. Coloured symbols (blue, yellow, orange and red) relate to escalation levels (1, 2, 3 and 4) from least (blue) to most (red) energy-demanding contests. (TIF) [file pone.0059774.s002.tif]

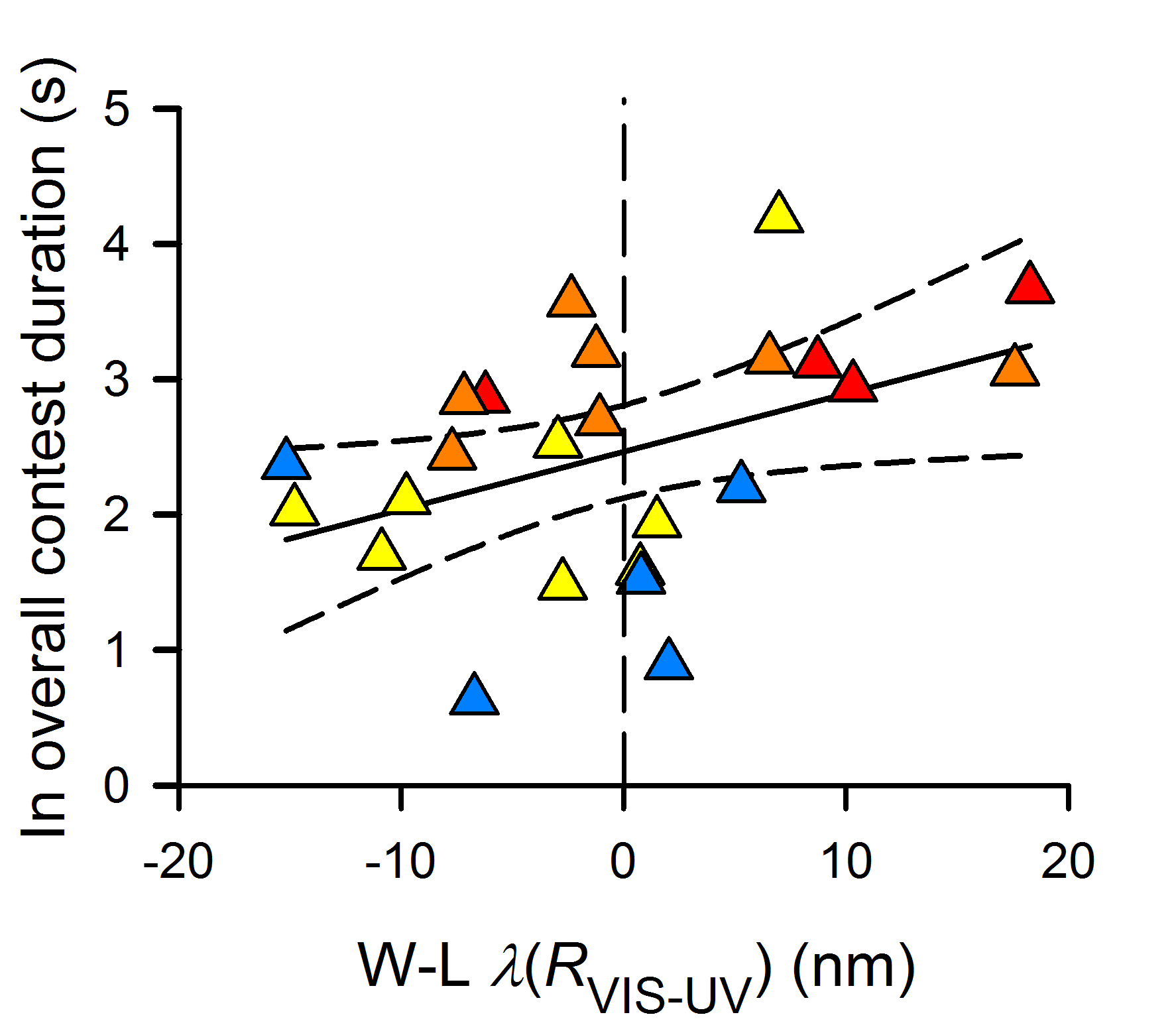

Supplement: Figure S3 — Influence of winners-losers asymmetry (W−L) in carapace (Δ) band separation on overall contest duration (natural log). Coloured symbols (blue, yellow, orange and red) relate to escalation levels (1, 2, 3 and 4) from least (blue) to most (red) energy-demanding contests. (TIF) [file pone.0059774.s003.tif]

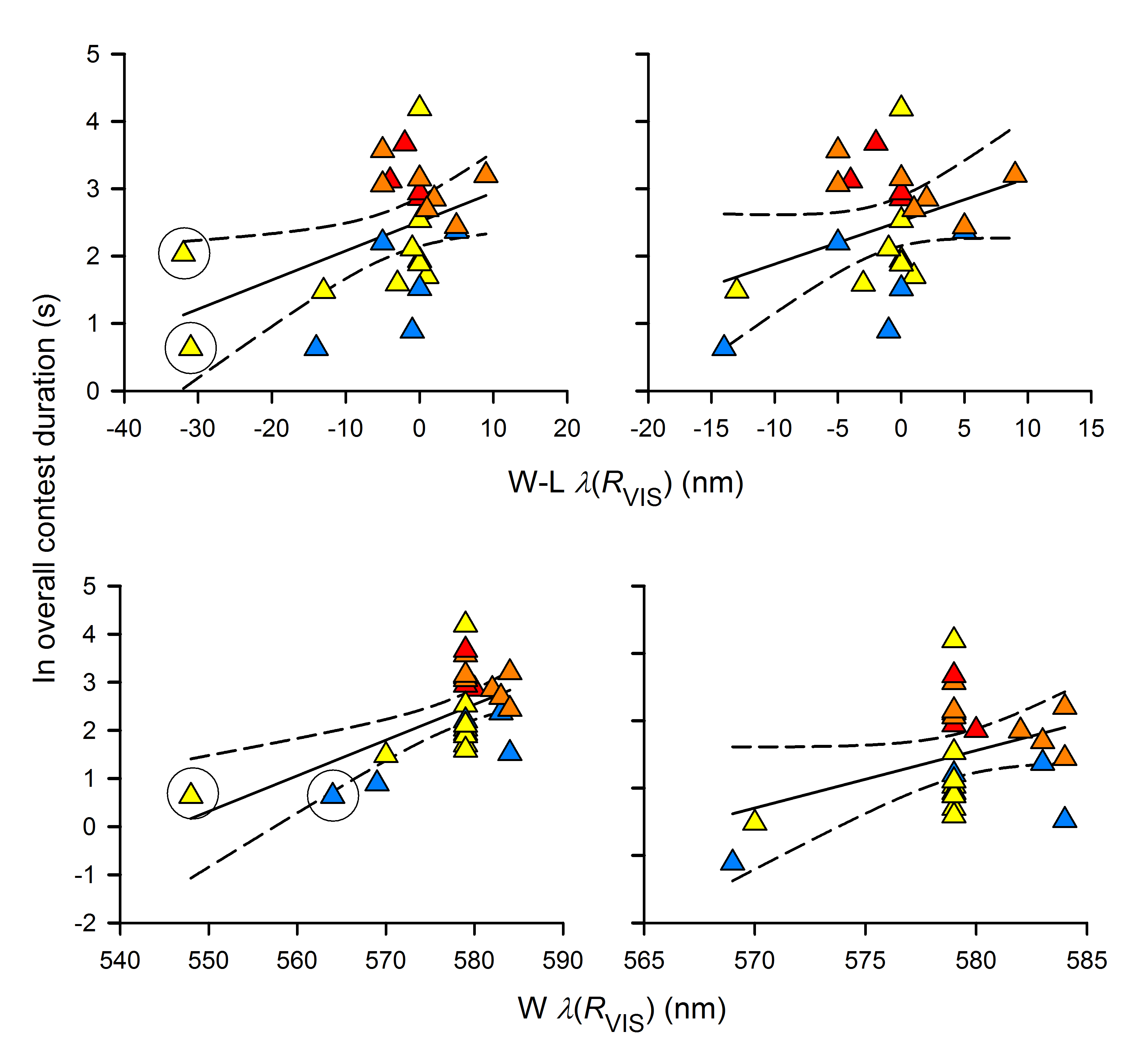

Supplement: Figure S4 — Influence of winners-losers asymmetry (W−L; top) and winners’ (W; bottom) carapace (Δ) VIS hue on overall contest duration and escalation, with presence (left) and absence (right) of data points where leverage values exceeded leverage critical values (circled points). Both rival asymmetry (R 2 = 0.13; P = 0.084) and winners’ (R 2 = 0.153; P = 0.066) carapace VIS hue did not predict overall contest duration and escalation after outliers (circled symbols) were removed. Outliers were identified using leverage critical values from the formula (3p−1)/n, where p and n refer to number of parameters and sample size, respectively. Coloured symbols (blue, yellow, orange and red) relate to escalation levels (1, 2, 3 and 4) from least (blue) to most (red) energy-demanding contests. (TIF) [file pone.0059774.s004.tif]

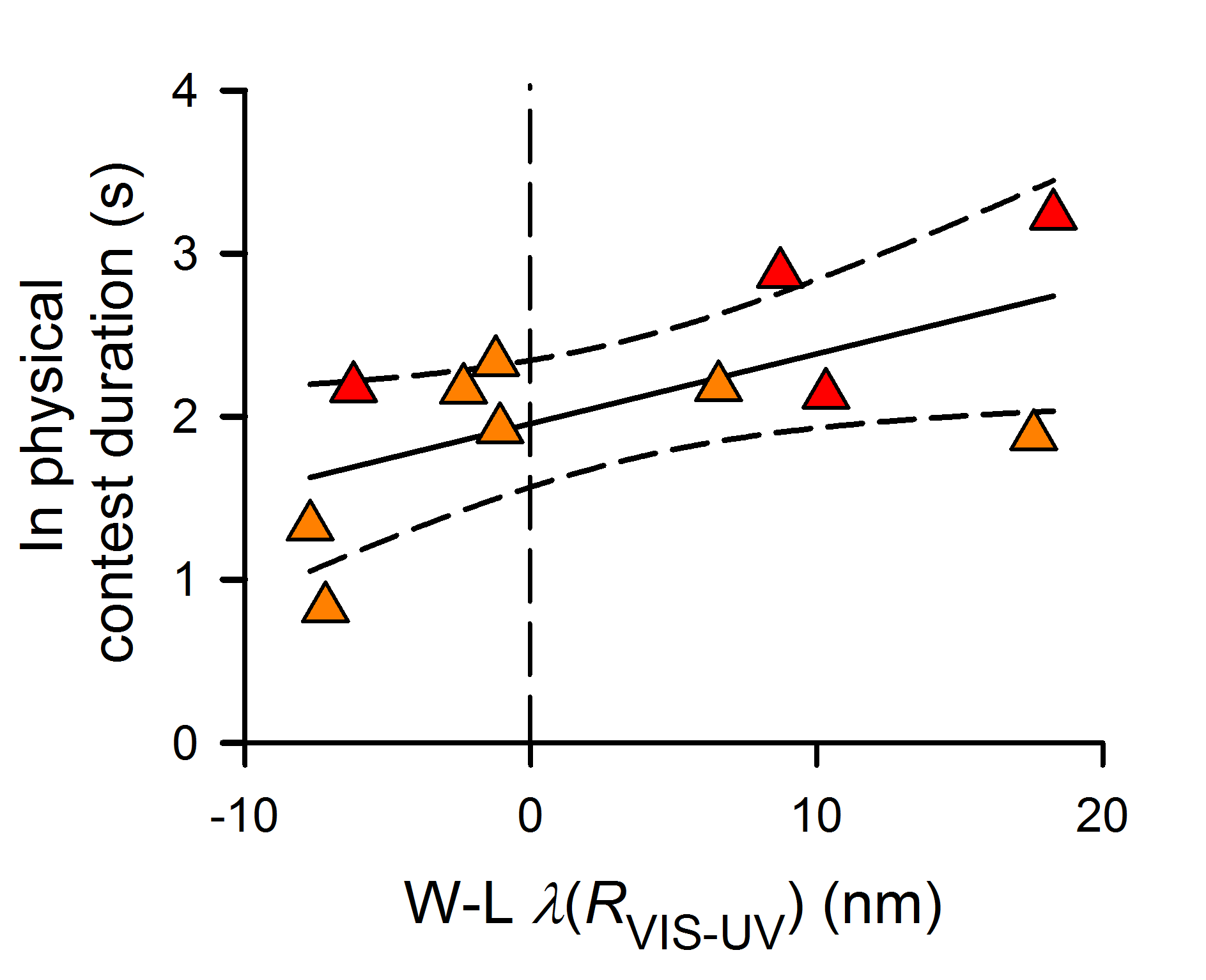

Supplement: Figure S5 — Influence of asymmetry (W−L) in carapace (Δ) band separation on duration of physical contests. Coloured symbols (orange and red) relate to escalation levels 3 and 4, respectively. (TIF) [file pone.0059774.s005.tif]

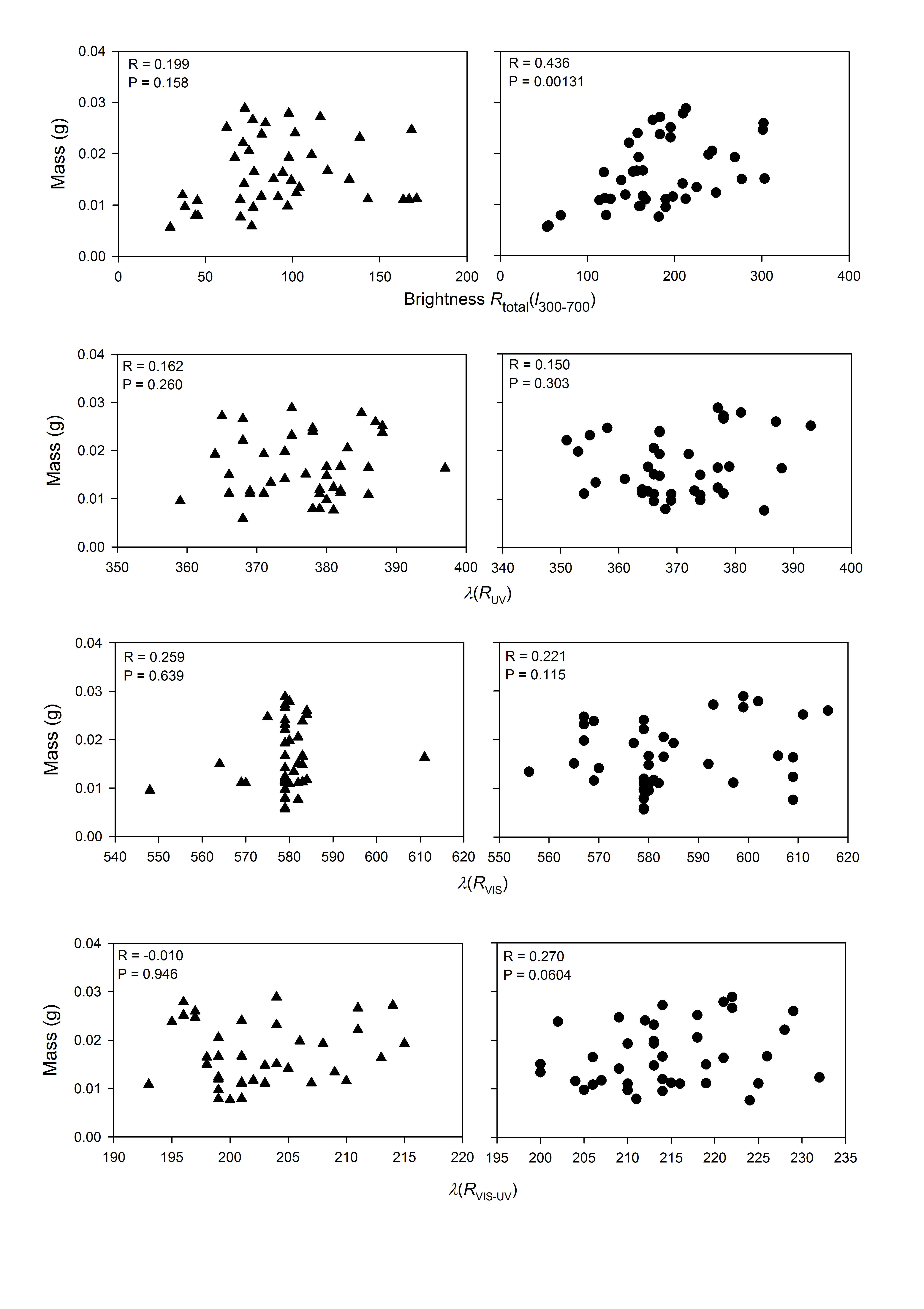

Supplement: Figure S6 — Correlations of carapace (Δ) or abdomen (○) colour traits with body mass of all individuals. Only abdomen total brightness exhibited a positive correlation with body mass. R: Spearman’s correlation coefficient. (TIF) [file pone.0059774.s006.tif]
