# Supplementary material for: UV-Green Iridescence Predicts Male Quality during Jumping Spider Contests
Source: PLoS One. 2013 Apr 3;8(4):e59774. doi: 10.1371/journal.pone.0059774 (PMC3616068; doi:10.1371/journal.pone.0059774)
Supplement: Table S1 — Differences in morphological and colour traits between initiators and non-initiators. (DOCX) [file pone.0059774.s007.docx]

| **Morphological & colour traits** | **Body part** | **Initiator (mean ± SE)** | **Non-initiator**  **(mean ± SE)** | **Statistics** | **Notes** |
| --- | --- | --- | --- | --- | --- |
| Body length  (mm) | NA | 63.538 ± 1.779 | 63.462 ± 1.839 | *t*_25_ = 0.129; *P* = 0.898 | N.S. |
| Mass  (g × 10^-2^) | NA | 1.558 ± 0.127 | 1.573 ± 0.128 | *t*_25_ = -0.799 ; *P* = 0.432 | N.S. |
| UV intensity  (area × 10^2^)  (arbitrary units) | Carapace | 10.764 ± 1.472 | 8.882 ± 1.522 | *t*_25_ = 0.982; *P* = 0.336 | N.S. |
|  | Abdomen | 15.668 ± 1.929 | 14.880 ± 2.081 | *t*_25_ = 0.338; *P* = 0.738 |  |
| VIS intensity  (area × 10^2^)  (arbitrary units) | Carapace | 86.172 ± 5.340 | 78.743 ± 5.714 | *Z* = -1.689; *P* = 0.094 | N.S. |
|  | Abdomen | 168.379 ± 9.502 | 162.266 ± 9.237 | *t*_25_ = 0.589; *P* = 0.561 |  |
| Total brightness  *R*_total_(λ_300-700nm_) (area×10^2^)  (arbitrary units) | Carapace | 96.937 ± 6.731 | 87.625 ± 7.127 | *Z* = -1.740; *P* = 0.084 | N.S. |
|  | Abdomen | 184.047 ± 11.202 | 177.146 ± 10.984 | *t*_25_ = 0.564; *P* = 0.577 |  |
| UV hue  λ(*R*_UV_)  (nm) | Carapace | 376.876 ± 1.465 | 375.691 ± 1.757 | *t*_23*_ = 1.049; *P* = 0.305 | N.S. |
|  | Abdomen | 370.822 ± 1.798 | 369.331 ± 2.150 | *t*_22**_ = 0.597; *P* = 0.556 |  |
| VIS hue  λ(*R*_VIS_)  (nm) | Carapace | 578.027 ± 1.463 | 580.307 ± 1.397 | *Z* = 0.698; *P* = 0.495 | N.S. |
|  | Abdomen | 585.770 ± 2.779 | 581.568 ± 2.856 | *t* = 1.045; *P* = 0.306 |  |
| Band separation  λ_VIS-UV_  (nm) | Carapace | 201.151 ± 1.038 | 204.752 ± 1.283 | *t*_23*_ = -2.102; *P* = 0.047 | Initiators exhibited smaller carapace band separation (fig. 1) |
|  | Abdomen | 215.233 ± 1.534 | 212.468 ± 1.729 | *t*_22**_ = 1.109; *P* = 0.279 |  |

*2 individuals did not exhibit prominent carapace UV hues

**3 individuals did not exhibit prominent abdomen UV hues.
