# Supplementary material for: UV-Green Iridescence Predicts Male Quality during Jumping Spider Contests
Source: PLoS One. 2013 Apr 3;8(4):e59774. doi: 10.1371/journal.pone.0059774 (PMC3616068; doi:10.1371/journal.pone.0059774)
Supplement: Table S2 — Differences in morphological and colour traits between winners and losers. (DOCX) [file pone.0059774.s008.docx]

| **Morphological & colour traits** | **Body part** | **Winner**  **(mean ± SE)** | **Loser**  **(mean ± SE)** | **Statistics** | **Notes** |
| --- | --- | --- | --- | --- | --- |
| Body length  (mm) | NA | 1.577 ± 0.127 | 1.554 ± 0.127 | *t*_25_ = 1.270; *P* = 0.216 | N.S. |
| Mass  (g × 10^-2^) | NA | 9.165 ± 1.797 | 9.256 ± 1.815 | *t*_25_ = 1.785; *P* = 0.086 | N.S. |
| UV intensity  (area × 10^2^)  (arbitrary units) | Carapace | 10.247 ± 1.500 | 9.399 ± 1.514 | *t*_25_ = 0.436; *P* = 0.667 | N.S. |
|  | Abdomen | 14.903 ± 1.912 | 15.646 ± 2.097 | *t*_25_ = -0.318; *P* = 0.753 |  |
| VIS intensity  (area × 10^2^)  (arbitrary units) | Carapace | 83.373 ± 5.065 | 81.542 ± 6.046 | *t*_25_ = 0.266; *P* = 0.792 | N.S. |
|  | Abdomen | 163.804 ± 8.928 | 16.684 ± 9.822 | *t*_25_ = -0.291; *P* = 0.773 |  |
| Total brightness  *R*_total_(λ_300-700nm_) (area×10^2^)  (arbitrary units) | Carapace | 93.620 ± 6.505 | 90.941 ± 7.441 | *t*_25_ = 0.308; *P* = 0.761 | N.S. |
|  | Abdomen | 178.707 ± 10.654 | 182.486 ± 11.595 | *t*_25_ = -0.305; *P* = 0.763 |  |
| UV hue  λ(*R*_UV_)  (nm) | Carapace | 374.825 ± 1.700 | 377.912 ± 1.425 | *t*_23*_ = -0.944; *P* = 0.355 | N.S. |
|  | Abdomen | 370.940 ± 1.627 | 369.207 ± 2.290 | *t*_22**_ = 0.689; *P* = 0.498 |  |
| VIS hue  λ(*R*_VIS_)  (nm) | Carapace | 577.160 ± 1.495 | 581.017 ± 1.344 | *Z* = 1.526; *P* = 0.131 | N.S. |
|  | Abdomen | 585.534 ± 2.644 | 581.805 ± 2.993 | *t*_25_ = 0.923; *P* = 0.365 |  |
| Band separation  λ_VIS-UV_  (nm) | Carapace* | 202.582 ± 1.364 | 203.203 ± 1.004 | *t*_23*_ = -0.226; *P* = 0.823 | N.S. |
|  | Abdomen* | 214.868 ± 1.645 | 212.848 ± 1.639 | *t*_22*_ = 0.811; *P* = 0.425 |  |

*2 individuals did not exhibit prominent carapace UV hues

**3 individuals did not exhibit prominent abdomen UV hues.
