# Supplementary material for: UV-Green Iridescence Predicts Male Quality during Jumping Spider Contests
Source: PLoS One. 2013 Apr 3;8(4):e59774. doi: 10.1371/journal.pone.0059774 (PMC3616068; doi:10.1371/journal.pone.0059774)
Supplement: Table S3 — Effects of carapace and abdomen colour traits on log-transformed overall contest duration. L, W, and W-L denote losers, winners, and winner-loser asymmetry, respectively. (DOCX) [file pone.0059774.s009.docx]

| **Colour traits** | | ***R*^2^** |  | ***b* ± SE** | ***F*** | ***dfs*** | ***P*** | **Notes** |
| --- | --- | --- | --- | --- | --- | --- | --- | --- |
| Carapace* | Total brightness  *R*_total_(λ_300-700nm_) (area×10^2^)  (arbitrary units) | 0.245 | W-L | −0.010 ± 0.004 | 7.791 | 1,24 | 0.010 | Carapace total brightness (normalized) asymmetry predicted overall contest duration (fig. 3, top) |
|  |  | 0.247 | L | 0.010 ± 0.005 | 4.583 | 1,23 | 0.043 | Both winners’ and losers’ carapace total brightness (normalized) predicted overall contest duration (fig. S2) |
|  |  |  | W | −0.011 ± 0.005 | 4.666 | 1,23 | 0.041 |  |
|  | Band separation  λ_VIS-UV_  (nm) | 0.195 | W-L | 0.043 ± 0.019 | 5.318 | 1,22 | 0.031 | Carapace band separation asymmetry predicted overall contest duration (fig. S3) |
|  |  | 0.249 | L | −0.006 ± 0.034 | 0.029 | 1,21 | 0.867 | Only winners’ carapace band separation predicted overall contest duration (fig. 4, top) |
|  |  |  | W | 0.067 ± 0.026 | 6.431 | 1,21 | 0.019 |  |
|  | UV hue  λ(*R*_UV_)  (nm) | 0.014 | W-L | −0.009 ± 0.017 | 0.312 | 1,22 | 0.582 | N.S. |
|  |  | 0.016 | L | 0.012 ± 0.027 | 0.204 | 1,21 | 0.656 |  |
|  |  |  | W | −0.008 ± 0.023 | 0.123 | 1,21 | 0.729 | N.S. |
|  | VIS hue  λ(*R*_VIS_)  (nm) | 0.199 | W-L | 0.043 ± 0.018 | 5.974 | 1,24 | 0.022** | N.S. (see fig. S4 for explanation) |
|  |  | 0.373 | L | −0.008 ± 0.023 | 0.136 | 1,23 | 0.715 |  |
|  |  |  | W | 0.075 ± 0.020 | 13.697 | 1,23 | 0.001** |  |
| Abdomen* | Total brightness  *R*_total_(λ_300-700nm_) (area×10^2^)  (arbitrary units) | 0.153 | W-L | −0.006 ± 0.003 | 4.340 | 1,24 | 0.048 | Abdomen total brightness asymmetry predicted overall contest duration (fig. 3, bottom) |
|  |  | 0.155 | L | 0.006 ± 0.003 | 3.587 | 1,23 | 0.071 |  |
|  |  |  | W | −0.005 ± 0.004 | 2.161 | 1,23 | 0.155 |  |
|  | Band separation  λ_VIS-UV_  (nm) | 0.109 | W-L | −0.024 ± 0.015 | 2.680 | 1,22 | 0.116 | N.S. |
|  |  | 0.135 | L | 0.007 ± 0.025 | 0.080 | 1,21 | 0.780 |  |
|  |  |  | W | −0.039 ± 0.024 | 2.673 | 1,21 | 0.117 |  |
|  | UV hue  λ(*R*_UV_)  (nm) | 0.005 | W-L | 0.005 ± 0.015 | 0.102 | 1,22 | 0.753 | N.S. |
|  |  | 0.012 | L | 0.000 ± 0.019 | 0.000 | 1,21 | 0.997 |  |
|  |  |  | W | 0.012 ± 0.025 | 0.242 | 1,21 | 0.628 |  |
|  | VIS hue  λ(*R*_VIS_)  (nm) | 0.024 | W-L | −0.007 ± 0.009 | 0.584 | 1,24 | 0.452 | N.S. |
|  |  | 0.025 | L | 0.006 ± 0.012 | 0.217 | 1,23 | 0.645 |  |
|  |  |  | W | −0.008 ± 0.014 | 0.358 | 1,23 | 0.556 |  |

*2 individuals did not exhibit prominent carapace UV hue; 3 individuals did not exhibit prominent abdomen UV hue

** Significant alpha values due to outliers.
