# Supplementary material for: UV-Green Iridescence Predicts Male Quality during Jumping Spider Contests
Source: PLoS One. 2013 Apr 3;8(4):e59774. doi: 10.1371/journal.pone.0059774 (PMC3616068; doi:10.1371/journal.pone.0059774)
Supplement: Table S4 — Effects of carapace and abdomen colour traits on log-transformed overall duration of physical contests. L, W, and W-L denote losers, winners, and winner-loser asymmetry, respectively. (DOCX) [file pone.0059774.s010.docx]

| **Colour traits** | | ***R*^2^** |  | ***b* ± SE** | ***F*** | ***dfs*** | ***P*** | **Notes** |
| --- | --- | --- | --- | --- | --- | --- | --- | --- |
| Carapace | Total brightness  *R*_total_(λ_300-700nm_) (area×10^2^)  (arbitrary units) | 0.001 | W-L | −0.001 ± 0.010 | 0.007 | 1,9 | 0.933 | N.S. |
|  |  | 0.157 | L | 0.007 ± 0.011 | 0.360 | 1,8 | 0.565 | N.S. |
|  |  |  | W | 0.020 ± 0.018 | 1.201 | 1,8 | 0.305 |  |
|  | Band separation  λ_VIS-UV_  (nm) | 0.392 | W-L | 0.043 ± 0.018 | 5.811 | 1,9 | 0.039 | Carapace band separation asymmetry predicted duration of physical contests (fig. S5) |
|  |  | 0.569 | L | −0.101 ± 0.037 | 7.712 | 1,8 | 0.024 | Only losers’ carapace band separation predicted duration of physical contests (fig. 4, bottom); higher standardized coefficients ***b*** in losers’ band separations |
|  |  |  | W | 0.022 ± 0.020 | 1.119 | 1,8 | 0.321 |  |
|  | UV hue  λ(*R*_UV_)  (nm) | 0.360 | W-L | −0.032 ± 0.014 | 5.064 | 1,9 | 0.051 | N.S. |
|  |  | 0.462 | L | 0.077 ± 0.040 | 3.744 | 1,8 | 0.089 |  |
|  |  |  | W | −0.011 ± 0.022 | 0.244 | 1,8 | 0.634 |  |
|  | VIS hue  λ(*R*_VIS_)  (nm) | 0.107 | W-L | −0.050 ± 0.048 | 1.080 | 1,9 | 0.326 | N.S. |
|  |  | 0.302 | L | −0.068 ± 0.091 | 0.561 | 1,8 | 0.475 |  |
|  |  |  | W | −0.191 ± 0.103 | 3.415 | 1,8 | 0.102 |  |
| Abdomen | Total brightness  *R*_total_(λ_300-700nm_) (area×10^2^)  (arbitrary units) | 0.012 | W-L | 0.001 ± 0.003 | 0.109 | 1,9 | 0.749 | N.S. |
|  |  | 0.077 | L | −0.002 ± 0.004 | 0.378 | 1,8 | 0.555 |  |
|  |  |  | W | −0.003 ± 0.006 | 0.230 | 1,8 | 0.644 |  |
|  | Band separation  λ_VIS-UV_  (nm) | 0.214 | W-L | −0.036 ± 0.023 | 2.446 | 1,9 | 0.152 | N.S. |
|  |  | 0.247 | L | 0.040 ± 0.026 | 2.403 | 1,8 | 0.160 |  |
|  |  |  | W | −0.034 ± 0.035 | 0.970 | 1,8 | 0.353 |  |
|  | UV hue  λ(*R*_UV_)  (nm) | 0.029 | W-L | −0.008 ± 0.016 | 0.268 | 1,9 | 0.617 | N.S. |
|  |  | 0.348 | L | 0.037 ± 0.020 | 3.393 | 1,8 | 0.103 |  |
|  |  |  | W | 0.022 ± 0.022 | 1.022 | 1,8 | 0.341 |  |
|  | VIS hue  λ(*R*_VIS_)  (nm) | 0.117 | W-L | −0.012 ± 0.011 | 1.188 | 1,9 | 0.304 | N.S. |
|  |  | 0.290 | L | 0.023 ± 0.013 | 3.073 | 1,8 | 0.118 |  |
|  |  |  | W | 0.005 ± 0.015 | 0.091 | 1,8 | 0.771 |  |
